# Mucilage facilitates root water uptake under edaphic stress: first evidence at the plant scale

**DOI:** 10.1093/aob/mcae193

**Published:** 2024-10-30

**Authors:** Mohanned Abdalla, Andrea Carminati, Gaochao Cai, Mutez Ali Ahmed

**Affiliations:** Root-Soil Interactions, School of Life Sciences, Technical University of Munich, Freising, Germany; Department of Horticulture, Faculty of Agriculture, University of Khartoum, Khartoum North, Sudan; Physics of Soils and Terrestrial Ecosystems, Department of Environmental Systems Science, ETH Zürich, Switzerland; School of Agriculture and Biotechnology, Shenzhen Campus of Sun Yat-sen University, Shenzhen, China; Root-Soil Interactions, School of Life Sciences, Technical University of Munich, Freising, Germany

**Keywords:** Drought, leaf water potential, plant hydraulics, rhizosphere, root exudates, soil–root interactions, transpiration

## Abstract

**Background and Aims:**

Mucilage has been hypothesized to soften the gradients in matric potential at the root–soil interface, thereby facilitating root water uptake in dry soils and maintaining transpiration with a moderate decline in leaf water potential. So far, this hypothesis has been tested only through simplified experiments and numerical simulations. However, the impact of mucilage on the relationship between transpiration rate (*E*) and leaf water potential (*ψ*_leaf_) at the plant scale remains speculative.

**Methods:**

We utilized an automated root pressure chamber to measure the *E*(*ψ*_leaf_) relationship in two cowpea genotypes with contrasting mucilage production. We then utilized a soil–plant hydraulic model to reproduce the experimental observations and inferred the matric potential at the root–soil interface for both genotypes.

**Key Results:**

In wet soil, the relationship between leaf water potential and transpiration rate (*E*) was linear for both genotypes. However, as the soil progressively dried, the *E*(*ψ*_leaf_) relationship exhibited non-linearity. The genotype with low mucilage production exhibited non-linearity earlier during soil drying, i.e. in wetter soil conditions (soil water content <0.36 cm^3^ cm^−3^) compared to the genotype with high mucilage production (soil water content <0.30 cm^3^ cm^−3^). The incidence of non-linearity was concomitant with the decline in matric potential across the rhizosphere. High mucilage production attenuated water potential diminution at the root–soil interface with increased *E*. This shows, for the first time at the plant scale, that root mucilage softened the gradients in matric potential and maintained transpiration in drying soils. The model simulations indicate that a plausible explanation for this effect is an enhanced hydraulic conductivity of the rhizosphere in genotypes with higher mucilage production.

**Conclusions:**

Mucilage exudation maintains the hydraulic continuity between soil and roots and decelerates the drop in matric potential near the root surface, thereby postponing the hydraulic limitations to transpiration during soil drying.

## INTRODUCTION

Plants are expected to experience increasing abiotic stresses due to climate change, including extreme events of drought, flooding, heat waves and freezing. Recent research in plant sciences aims to understand plant responses to these intensifying stresses and the underlying physiological mechanisms (McDowell *et al.*, 2022; Vereecken *et al.*, 2022; Verslues *et al.*, 2022). Among these stresses, drought episodes are one of the major abiotic stresses that not only limit crop productivity in many regions worldwide (Madadgar *et al.*, 2017) but also drive widespread tree mortality (Choat *et al.*, 2018; Brodribb *et al.*, 2020).

Soil drying diminishes the pool of water available for plants, limiting root water uptake and transpiration fluxes. Plants’ adaptations to contrasting environments include diverse strategies that enable plants to tolerate unfavourable conditions. In actively transpiring plants, water flows in the soil–plant–atmosphere continuum following gradients in water potential. Water fluxes are hindered by a series of resistances along the continuum. Hydrated soils have great capability to transport water to the roots, but, as soil dries, its hydraulic conductance declines by several orders of magnitude, especially around roots, causing large gradients in water potential at the root–soil interface (Gardner, 1960; Passioura, 1980; Draye *et al.*, 2010). This water potential drop in the rhizosphere results in non-linearity between transpiration rate and leaf water potential (Sperry and Love, 2015; Carminati and Javaux, 2020). In other words, to maintain a certain transpiration rate, leaf water potential needs to decline as soil dries, thus shifting the linear relationship between transpiration and leaf water potential observed in wet soil to a non-linear one (Carminati and Javaux, 2020; Abdalla *et al.*, 2021; Javaux and Carminati, 2021). However, plants respond by closing their stomata to avoid an excessive decline in leaf water potential (Sperry and Love, 2015; Carminati and Javaux, 2020; Abdalla *et al.*, 2022*a*).

The increase in soil–root hydraulic resistance, as a consequence of water potential decrease in soil, has been reported as the main hydraulic limitation triggering stomatal closure (North and Nobel, 1991, 1994; Carminati and Javaux, 2020; Rodriguez‐Dominguez and Brodribb, 2020; Abdalla *et al.*, 2021, 2022*a*; Bourbia *et al.*, 2021). These studies collectively highlight the pivotal role of the soil–root system in plant water use regulation. Moreover, modulating rhizosphere traits has been proposed as a successful means of adaptation to improve resource acquisition under scarcity conditions, e.g. phosphorus and water uptake, and mitigate adverse effects of abiotic stressors (Munns and Tester, 2008; van Zelm *et al.*, 2020; Karanja *et al.*, 2021; Püschel *et al.*, 2021; Abdalla *et al.*, 2022*b*; Cai and Ahmed, 2022; Koehler *et al.*, 2023; Zhang *et al.*, 2023). Previous rhizosphere research focused on contributions of root hairs (Carminati *et al.*, 2017*b*; Cai *et al.*, 2021; Marin *et al.*, 2021) and arbuscular mycorrhizal fungi (Augé *et al.*, 2015; Bitterlich *et al.*, 2018; Abdalla and Ahmed, 2021; Kakouridis *et al.*, 2022) in root water uptake and plant water status. Similarly, a number of studies (Guinel and McCully, 1986; Watt *et al.*, 1993; McCully and Boyer, 1997; Ahmed *et al.*, 2014) including mathematical simulations (Carminati *et al.*, 2011) have directed research towards understanding the pivotal role of root mucilage in root water uptake. However, our understanding of the role of root mucilage in the regulation of plant water use under drought remains incomplete, especially at the plant scale.

By facilitating the acquisition of soil resources, root exudates have been considered as a key adaptation feature of plants growing in different agroecological zones (Czarnes *et al.*, 2000; Kroener *et al.*, 2018; Nazari *et al.*, 2020). Mucilage, a polysaccharide substance secreted at the root tip, was shown to alter the rhizosphere hydraulic properties. Current efforts have attempted to understand its physicochemical properties and how they alter and/or buffer rapid oscillation in water dynamics in the vicinity of the root (Carminati *et al.*, 2010, 2017*a*; Kroener *et al.*, 2014; Naveed *et al.*, 2019). Further investigations explored ways by which mucilage increases soil water-holding capacity, water transport and soil water repellency during drying and rewetting (Czarnes *et al.*, 2000; Kroener *et al.*, 2014, 2018; Ahmed *et al.*, 2016; Benard *et al.*, 2018). Mucilage was suggested to facilitate water fluxes in drying soils, using an artificial root analogy (Ahmed *et al.*, 2014). The authors demonstrated that mucilage increased rhizosphere water content, thereby facilitating root water uptake in dry soil (Ahmed *et al.*, 2014). Similarly, using modelling, Carminati *et al.* (2011) showed that mucilage may favour water availability to the root by increasing the unsaturated hydraulic conductivity of the rhizosphere (Carminati *et al.*, 2011). Despite these efforts to explore the role of mucilage in root water uptake, no *in situ* evidence illustrates how mucilage can influence the gradients in matric potential at the root–soil interface and hence the relationship between transpiration rate and leaf water potential in drying soils.

**Fig. 1.**
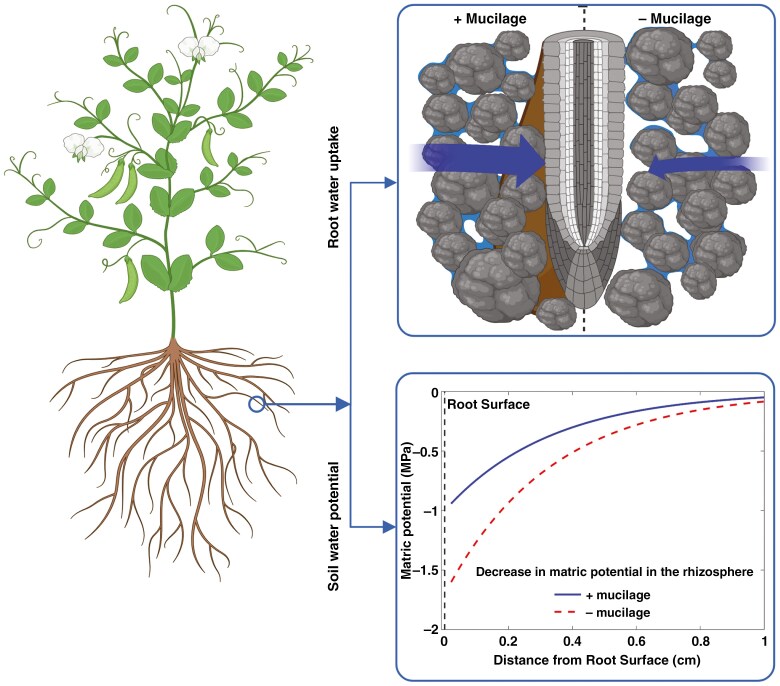
Illustration of the anticipated effects of contrasting root mucilage production on soil–root hydraulics. Without mucilage, the soil matric potential will drop steeply at the root surface, as indicated by the red arrow, which hinders root water uptake (blue arrow). On the other hand, high mucilage concentration at the root surface will maintain the hydraulic conductance between root and soil, and attenuate the decline in matric potential at the root surface (red arrow), hence maintaining root water uptake in drying soils (blue arrow). The figure was made in BioRender: Abdalla, M. (2024) https://BioRender.com/s66t170.

Here, we tested whether mucilage exudation facilitates water uptake in cowpea plants growing in loamy soil. We hypothesized that higher mucilage exudation attenuates the drop in matric potential at the root surface in dry soils (Fig. 1). In contrast, the decreases in matric potential across the rhizosphere will be much steeper in plants with low mucilage production (Fig. 1). The rationale is that mucilage influences the soil hydraulic conductivity under both saturation conditions (*K*_*sat*_) and its declining rate in response to decreasing soil matric potential (referred to as unitless τ, after Brooks and Corey, 1966). We experimentally tested our hypothesis, using two cowpea genotypes, with one genotype producing more root mucilage, by a factor of seven compared to the other genotype (Sun *et al.*, 2015*a*, *b*). We used a root pressure chamber system to measure the relationship between transpiration rate and leaf xylem water potential, similar to the pivotal work by Passioura (1980). The measurements were then compared to simulations that were generated using the soil–plant hydraulic model of Carminati and Javaux (2020). We further used this model to explore the effects of mucilage on soil hydraulic properties, root water uptake, and the relationship between transpiration rate and leaf water potential.

## MATERIALS AND METHODS

### Soil and plant preparation

We used two cowpea genotypes (*Vigna unguiculata* L.) with contrasting root mucilage production (Sun *et al.*, 2015*a*, *b*). Mucilage production differed by a factor of 7 between the two genotypes under investigation, IT and CB, namely 3.4 ± 0.8 (IT) and 0.5 ± 0.3 mg (CB) dry weight mucilage per gram of dry weight root, for the genotype with high mucilage and low mucilage production, respectively, as reported by Sun *et al.* (2015*b*). Seeds were treated with H_2_O_2_ for 10 min for surface sterilization, rinsed with distilled water three times and placed in Petri dishes onto a moist filter paper. Germinated seeds were planted in polyvinyl chloride (PVC) cylindrical pots (7 cm inner diameter and 30 cm height). The PVC pots had five holes on their sides, with a diameter of 5 mm, to enable the measurements of soil water content. An aluminium disc (0.8 cm thickness) was used to cover the pot using rubber glue (Teroson, Henkeln, Germany). The disc had a central aperture (1.4 cm in diameter), in which one germinated seed was placed per pot. This special set-up is needed to facilitate pressurizing roots and soil in intact cowpea plants. The PVC pots were filled with loamy soil with components of silt, sand and clay (33.2, 47.7 and 19.1 %, respectively). The hydraulic properties, i.e. soil water retention curves and unsaturated hydraulic conductivity curves, of the loamy soil are reported in Supplementary Data Fig. S1 and Vetterlein *et al.* (2021). Soil moisture content was measured using a time domain refractometer (TDR; E-Test, Luben, Poland), and the corresponding matric potentials were obtained through water retention curve (Fig. S1). Soil hydraulic parameters were used in the soil plant hydraulic model and solving Richard’s equation (Note S1).

Plants were grown at day–night temperatures of 22–18 °C and 12 h of photoperiod with a photosynthetic photon flux density (PPFD) of 350 µmol m^−2^ s^−1^ for 3 weeks. Thereafter, a rigid glue (Uhu Plus Endfest 300, Bühl, Germany) was applied to seal the aperture around the plant collar and the previously described aluminium disc that covers the pot to facilitate root pressurization (Abdalla *et al.*, 2021). Leaves were imaged to determine leaf area using ImageJ 1.50e (https://imagej.net). After the termination of the experiments, roots were extracted from the soil, washed and scanned (using an Epson STD 4800 scanner). Total root length was measured using WinRhizo software (Regent Instruments Inc., Quebec, Canada).

### Transpiration rate and balancing pressure

A root pressure chamber system (RPCS) was used to simultaneously measure transpiration rate (*E*) and the corresponding balancing pressure (*P*) in intact cowpea plants. The RPCS was re-introduced by Cai *et al.* (2020*b*) after Passioura (1980). Briefly, the major components of the RPCS are a root pressure chamber, cuvette and main controller (Supplementary Data Fig. S2). The pot was placed inside the root pressure chamber to obtain *P*, while the shoots were enclosed inside the cuvette to obtain *E* simultaneously and non-destructively, with high temporal resolutions. The cuvette was equipped with four groups of light-emitting diode (LED) lamps to provide PPFD around shoots. The PPFD was adjusted stepwise (to mimic the natural increment of light from predawn to midday) to increase *E*. In other words, *E* was altered by increasing PPFD from 0 to 100, 200, 400, 600, 800 and 1000 µmol m^−2^ s^−1^. Relative humidity, temperature and airflow rate were measured, every 10 s, from the inlet and the outlet of the cuvette. Hence, *E* was determined by multiplying the airflow rate with the difference between ingoing and outgoing humidity (Cai *et al.*, 2020*b*).

During the measurements, leaf xylem water potential (*ψ*_leaf_) was kept at atmospheric pressure, i.e. equal to zero, which was facilitated by an automated meniscus system equipped in the RPCS. To do so, the root and soil were pressurized to bring a droplet on a cut at the leaf petiole. The stability of the droplet was ensured using an automated meniscus system that was made of fine capillary tubing (diameter = 0.5 mm) and a laser-based sensor. We considered that the applied pressure was equal to the tension inside the leaf xylem that had not been pressurized by a stable reading of the meniscus system (± 2 mm or less) for 10 min (Passioura, 1980; Cai *et al.*, 2020*b*; Abdalla *et al.*, 2021). The applied pressure was then inferred as the balancing pressure (*P*). This was initially done at low PPFD (i.e. 0 µmol m^−2^ s^−1^) and repeated stepwise at each level of PPFD (up to 1000 µmol m^−2^ s^−1^) (Abdalla *et al.*, 2022*a*). Each step lasted for 30–60 min, and each plant was measured three times (i.e. 3 d) at different levels of soil dryness, i.e. wet, relatively dry and dry (the corresponding soil water potentials are provided in the Results section).

For each soil water content, we interpolated the slope of the first four data points (0, 100, 200 and 400 µmol m^−2^ s^−1^) to create a linear relationship, assuming no non-linearity in the *E*(*ψ*_leaf_) relationship. We then calculated the deviation of measured *P* from this interpolated linear relationship at each data point. This deviation represents the gradients in water potential across the rhizosphere at each *E*, given that the leaf water potential was kept very high, i.e. at atmospheric pressure by design.

It is noteworthy that this method provides the hydrostatic (pressure potential) component of water potential and ignores the osmotic one. The latter causes an offset in the relationship between *P* and *E*, which can be calculated using a mechanistic soil–plant model (Carminati and Javaux, 2020). In sum, to measure the relationship between *P* and *E*, plants were placed inside the RPCS, the pot inside the pressure chamber and the shoot inside the cuvette. We boosted *E* by increasing PPFD stepwise (0, 100, 200, 400, 800 and 1000 µmol m^−2^ s^−1^), while determining *P* simultaneously at each PPFD step. This procedure was repeated for each plant during soil drying that occurred naturally during the days of measurements. Three plants were measured per genotype at three levels of soil dryness from wet (first day) to dry (third/fourth day) and the corresponding soil water content/potential was measured/calculated and is reported in the results section and Supplementary Data Fig. S1.

### Soil–plant hydraulic model

The soil–plant model introduced by Carminati and Javaux (2020) was used to simulate the water potential gradients across the soil–plant system. The objective was to reproduce the dynamic changes in water potential in the soil–root system in response to increasing *E* at a given soil water potential. More details of this model are described in Carminati and Javaux (2020), Abdalla *et al*. (2022*a*) and Supplementary Data Note S1 and have been used elsewhere (Cai *et al.*, 2020*a*, 2021, 2022*a*; Hayat *et al.*, 2020; Abdalla *et al.*, 2021, 2022*b*). Briefly, the model calculates a series of resistances across the soil–plant system and assumes a steady-state water flow. Water flow in the soil was simulated assuming a radial geometry and a uniform root water uptake into a fraction of the total root length, hereafter called effective root length. Water potential was calculated in the soil, at the soil–root interface and in the leaf xylem (Note S1). We briefly describe the model here.

The Buckingham–Darcy law was used to describe the radial water flow in soil toward the root surface:


$$\begin{array}{l} q=-K_{s}\left(\psi_{m}\right)\frac{\partial \psi_{m}}{\partial r}\; \end{array}$$ (1)

where *q* is the water flux (cm s^−1^); *K*_*s*_ is the soil hydraulic conductivity (cm^2^ s^−1^ hPa^−1^), which is a function of the soil matric potential *ψ*_*m*_ (hPa); *r* is the radial distance (cm); and $\frac{\partial \psi_{m}}{\partial r}$ is the gradient in matric potential. Note that when the soil matric potential is expressed in unit heads (cm, 1 hPa ≈ 1 cm), *K*_*s*_ has units of (cm s^−1^). We used this unit throughout the text when describing soil water flow.

*K*_*s*_ was parameterized according to the Brooks and Corey model:


$$\begin{array}{l} K_{s}\left(\psi_{m}\right)=\;K_{sat}\;{\left(\frac{\psi_{m}}{\psi_{0}}\right)}^{\tau } \end{array}$$ (2)

where *K*_*sat*_ is the saturated hydraulic conductivity of the soil (cm s^−1^), *ψ*_0_ is the soil air entry value (cm) and τ is a dimensionless fitting parameter.

The boundary conditions were expressed as follows:


$$\begin{array}{l} q\left(r_{0}\right)=\frac{E}{2\pi r_{0}L} \end{array}$$ (3)

Water flow in the root is given by:


$$\begin{array}{l} E=-K_{root}\left(\psi_{root\_xylem}-\psi_{root\_soil}\right) \end{array}$$ (4)

where *E* is the water flow in the root equal to the transpiration rate (cm^3^ s^−1^), $\psi_{root\_soil}$ is the water potential at the root–soil interface (here converted to MPa, where 1 MPa ≈ 10 000 cm), $\psi_{root\_xylem}$ is the water potential at the xylem collar (MPa) and $K_{root}$ (cm^3^ s^−1^ MPa^−1^) is the root hydraulic conductance. Please refer to Supplementary Data Note S1 for more details about the model and also to Carminati and Javaux (2020). Parameters used to reproduce the relationship between transpiration rate and leaf water potential are provided in Table S1.

### Inverse modelling

We inversely modelled the relationship between transpiration rate and *ψ*_leaf_ to obtain the unsaturated hydraulic conductivity near roots with high and low mucilage genotypes. Transpiration rate, soil water potential and leaf xylem water potential (assumed to correspond to minus the balancing pressure) were fitted by varying the parameters *K*_*sat*_ and *τ* (see eqn 2). Both parameters were used in the inverse modelling to obtain rhizosphere hydraulic conductance after fixing other model parameters (Supplementary Data Table S1). The obtained results from the inverse modelling of unsaturated hydraulic conductivity with low and high mucilage were compared to the measured unsaturated hydraulic conductivity in unplanted soil (no mucilage; figures are provided in the Results section). In the model we kept root length equal for the two genotypes. This assumption was justified by the independent measurements of roots, but it definitely affects the accuracy of the estimation from the inverse modelling. In this sense, it might also be that the effect of mucilage is to increase the effective fraction of the root surface that extracts water from the soil. Therefore, the reported hydraulic conductivity has to be considered as an effective property, rather than the actual conductivity of specific segments of the rhizosphere.

### Statistical analyses

Two genotypes were statistically compared to evaluate significant differences in biomass, namely leaf area, root length and root diameter. A t-test was used for evaluation and statistical significance was considered at *P* < 0.05. A linear segmented regression model was performed to test the incidence of non-linearity in the relationship between transpiration rate and leaf water potential during soil drying. The relationships were compared per genotype–soil moisture combinations using the ‘segmented’ package in R (R Core Team, 2019; Muggeo, 2024), which allows the detection of breakpoints where the slope of the relationship between transpiration rate and leaf water potential changes. The presence of significant breakpoints was assessed by evaluating their confidence intervals and standard errors. MATLAB software (Math Works Inc., USA) was used to perform statistical analyses and modelling simulations.

## RESULTS AND DISCUSSION

### Comparative morphological analysis of cowpea genotypes

Two genotypes of cowpea with contrasting mucilage production were investigated to understand the role of mucilage in water uptake. We observed no significant differences in leaf area [765.5 ± 215 cm^2^ (s.d.), 1024.7 ± 123 cm^2^] between genotypes with low or high mucilage production, respectively (*P* = 0.0711; Supplementary Data Fig. S3). Likewise, root length (4151 ± 2550 cm, 3872 ± 1230 cm, *P* = 0.902) and root diameter (0.46 ± 0.021 cm, 0.45 ± 0.004 cm, *P* = 0.439) did not differ significantly between the two genotypes (Fig. S3).

### Relationship between transpiration rate and leaf xylem water potential

**Fig. 2.**
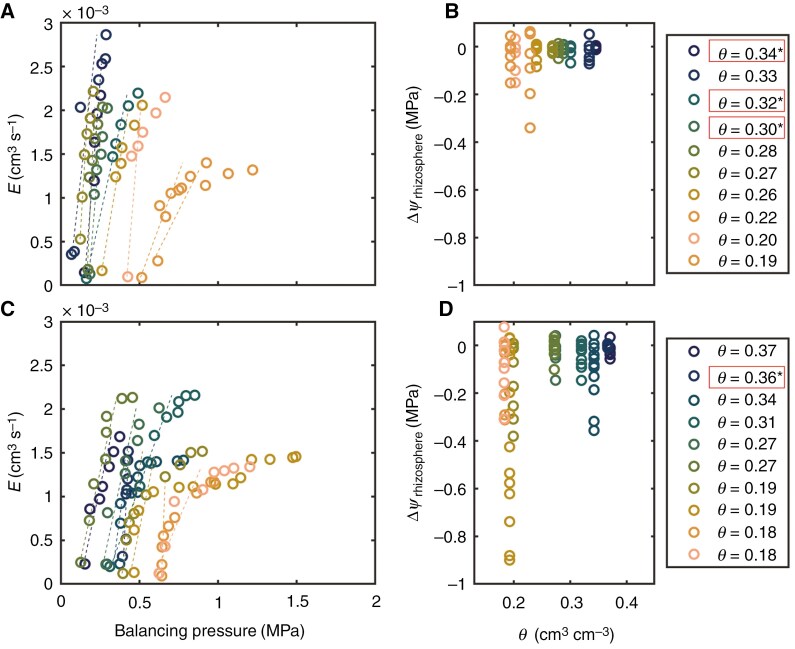
Relationship between balancing pressure and transpiration rate in two cowpea genotypes with contrasting mucilage production during soil drying. (A) Genotype with high mucilage production. (C) Genotype with low mucilage production. Open symbols represent direct measurements, while dotted lines indicate linear interpolations based on the slope of the first four data points. The relationship is linear in wet soil and exhibits non-linearity as soil progressively dries. B and D show water potential decrease across the rhizosphere, in response to increasing transpiration rate, for the genotype with high mucilage (B) and for the genotype with low mucilage production (D). Declining soil water content (*θ*, cm^3^ cm^−3^) is indicated by the colour map. Asterisks denote linear relationships based on a segmented linear model.

**Fig. 3.**
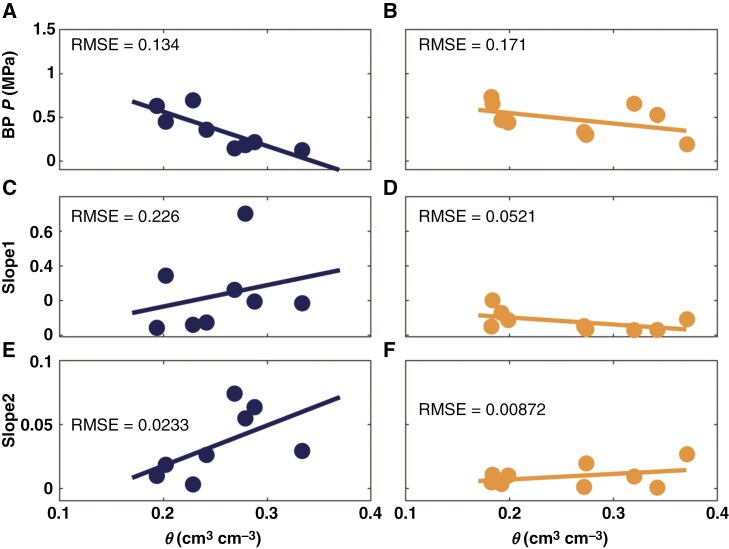
Coefficient estimates of segmented linear regression of the relationship between transpiration rate and balancing pressure (*P*) for the high mucilage genotype (A, C and E) and low mucilage genotype (B, D and F). Both A and B depict the breakpoint (BP) when the relationship changes from linear to non-linear. The slope of the relationship before the BP (Slope1; cm^3^ s^−1^ MPa^−1^) is shown in C and D, while the slope of the relationship after the BP (Slope2; cm^3^ s^−1^ MPa^−1^) is shown in E and F.

In wet soil, the relationship between the balancing pressure (*P*, −*ψ*_leaf_) and transpiration rate (*E*) was linear for both cowpea genotypes. However, as the soil progressively dried, the *E*(*ψ*_leaf_) relationship exhibited non-linearity (Fig. 2A, C). This result is in line with previous findings in monocot crops (Passioura, 1980; Carminati *et al.*, 2017*b*; Hayat *et al.*, 2020; Cai *et al.*, 2021) and herbaceous species (Abdalla *et al.*, 2021, 2022*a*). For plants with high mucilage production, the relationship became non-linear with a soil water content (*θ*) of <0.30 cm^3^ cm^−3^ (Fig. 2A, B). However, in plants with low mucilage production, the non-linearity occurred at higher soil water content (*θ* < 0.36 cm^3^ cm^−3^; Fig. 2C; Supplementary Data Fig. S4). The incidence of non-linearity was statistically indicated by the linear segmented regression model (Fig. 2; Fig. S4). Additionally, in plants with low mucilage, the linearity is lost at relatively lower transpiration rates: ~ 2 × 10^−3^ in wet soil, and 1–1.5 × 10^−3^ cm^3^ s^−1^ in dry soil. On the other hand, in plants with high mucilage production, the non-linearity occurred at lower soil moisture content and relatively higher transpiration rate (Fig. 2). The segmented linear model indicated that plants with high mucilage production linearized the relationship between *E* and *P* (Fig. 3; Fig. S4). This effect is evident from the higher slope of the second segment – i.e. after the onset of hydraulic non-linearity – in plants with high mucilage [root mean squared error (RMSE) = 0.0233; Fig. 3E] compared to plants with low mucilage production (RMSE = 0.00872; Fig. 3F). In the genotype with low mucilage, the slope after the breakpoint in the *E*(*P*) relationship is particularly low (Slope2; Fig. 3F), indicating a strong loss in conductivity with increasing transpiration rate.

### Mucilage impacting water potential gradients across the rhizosphere

In plants with high mucilage production, water potential propagation across the rhizosphere did not fall below −0.07 MPa in relatively wet soil (*θ* ≥ 0.30 cm^3^ cm^−3^; Fig. 2) and reached at most −0.15 MPa in dry soil (*θ* ≤ 0.20 cm^3^ cm^−3^; Fig. 2B). In contrast, in the low mucilage genotype, water potential decrease across the rhizosphere reached −0.15 MPa already in relatively wet soil (*θ* ≥ 0.30 cm^3^ cm^−3^; Fig. 2D), and it dropped further to −0.9 MPa as the soil progressively dried (*θ* ≤ 0.20 cm^3^ cm^−3^). The results support our hypothesis that mucilage exudation maintains the hydraulic continuity between soil and root and decelerates water potential drop in the soil near the root surface, which postpones hydraulic limitations to transpiration in dry soil. Indeed, declining soil–root hydraulic conductance has been proposed as the primary driver of stomatal closure (Carminati and Javaux, 2020; Rodriguez‐Dominguez and Brodribb, 2020; Abdalla *et al.*, 2021, 2022*a*; Bourbia *et al.*, 2021). Alteration of soil hydraulic conductance has been documented to influence plant water use regulation during soil drying (Hacke *et al.*, 2000; Carminati and Javaux, 2020; Javaux and Carminati, 2021; Abdalla *et al.*, 2022*a*; Cai *et al.*, 2022*b*). The underlying premise is that water potential loss in the soil around the root has been proposed as the trigger of disproportionality between declining water potential and water fluxes (Carminati and Javaux, 2020; Abdalla *et al.*, 2021, 2022*a*). The steep decline in root–soil water potential in response to increasing transpiration rate shown in the genotype with low mucilage production provides clear evidence that mucilage postpones the decline in water potential in the rhizosphere and maintains water fluxes from dry soils (Fig. 2). This observation implies that variations in soil–root hydraulics are initiated in early stages of drying in the rhizosphere, rather than the root, and mucilage buffers the adverse impacts of declining soil hydraulic conductance on soil–plant hydraulics. Previous studies, using mathematical modelling, proposed a similar concept that mucilage alters rhizosphere water dynamics and hydraulic conductance (Carminati, 2012). In other words, mucilage increases unsaturated soil hydraulic conductivity and rhizosphere water capacity that restore a higher matric potential at the root surface, which subsequently enhances root water uptake (Carminati *et al.*, 2011, 2017*a*; Ahmed *et al.*, 2014).

### Mucilage impacting soil–plant hydraulics, rhizosphere dynamics and plant water use

**Fig. 4.**
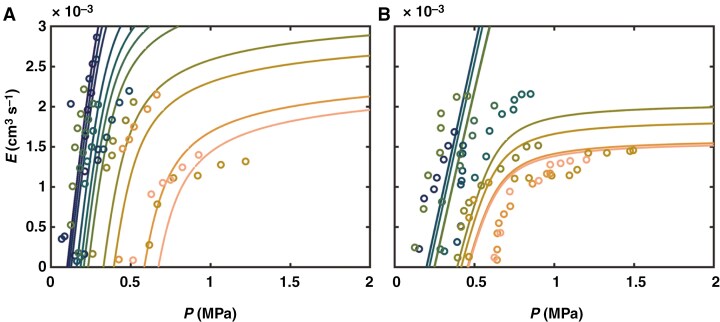
Relationship between balancing pressure (*P*) and transpiration rate (*E*) as reproduced by the soil–plant hydraulic model for the genotype with high mucilage production (A) and genotype with low mucilage production (B). Solid lines stand for modelling simulations and symbols for data measurements.

The *E*(*ψ*_leaf_) relationship was reproduced by the soil–plant hydraulic model at different soil water potentials (Fig. 4). The slope of the relationship in wet soil conditions gives the plant hydraulic conductance (or the reciprocal of plant hydraulic resistance), being 0.911 × 10^−6^ cm^3^ s^−1^ hPa^−1^ for the genotype with low mucilage and 1.87 × 10^−6^ cm^3^ s^−1^ hPa^−1^ for the genotype with high mucilage. Our inverse modelling showed an offset between measured and simulated predawn leaf water potential in the low mucilage genotype in dry soils (Fig. 4B). A similar offset has been reported recently in other crops (Cai *et al.*, 2020*a*, 2021; Abdalla *et al.*, 2022*a*, *b*). In this study, we assumed equal offset in both genotypes; however, the offset was greater in the genotype with low mucilage production and was not fully captured by the model (Fig. 4B). One possible explanation is the differences in osmotic potential between soil and plant (Donovan *et al.*, 2001; Zhang *et al.*, 2021), which might be caused by salt accumulation at the root surface during root water uptake (Stirzaker and Passioura, 1996; Abdalla *et al.*, 2022*b*). Our modelling result suggests interactions between mucilage and salt accumulation at the root surface during soil drying, which requires further investigation.

**Fig. 5.**
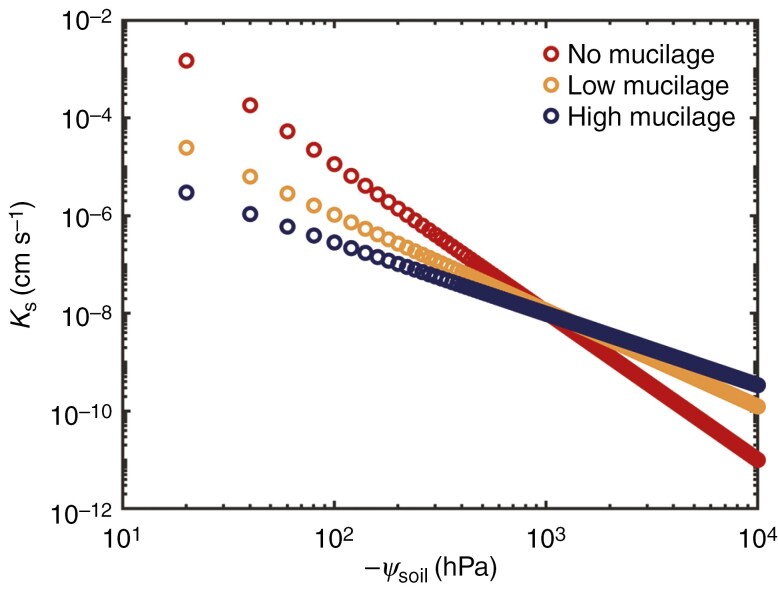
Soil hydraulic conductivity (*K*_*s*_) curve fitted by the Brooks and Corey model. The soil–plant hydraulic model predicted *K*_*s*_ in the rhizosphere during soil drying. The genotype with high mucilage production has decreased saturated hydraulic conductivity but higher unsaturated conductivity in more negative soil matric potential (*ψ*_soil_) compared to the other genotype and unplanted soil (referred to as no mucilage).

We used the soil–plant hydraulic model to predict the effective hydraulic conductivity of the rhizosphere. By fitting the *E*(*ψ*_leaf_*, ψ*_soil_) relationships, we found that the genotype with high mucilage production had lower rhizosphere hydraulic conductivity in saturated soil conditions, but it had a higher hydraulic conductivity at more negative matric potential (Fig. 5), which explains the more moderate decline in water potential across the rhizosphere of the plants with high mucilage content. This result is consistent with the reported capability of mucilage to maintain higher rhizosphere hydraulic conductivity at more negative soil matric potential. Our results are in line with previous studies that showed mucilage reduces saturated soil hydraulic conductivity and increases unsaturated hydraulic conductivity (Carminati, 2012; Benard *et al.*, 2019). Note that for both genotypes we had to vary the properties of the soil compared to unplanted soil, more markedly for the genotype with high mucilage production (Fig. 5). This might be explained by the presence of root hairs, which were shown to attenuate gradients in water potential across the rhizosphere (Carminati *et al.*, 2017*b*; Duddek *et al.*, 2023), and in general by the fact that rhizosphere and bulk soil probably differ from each other.

Note that the rhizosphere conductivities reported in Fig. 5 have to be considered as effective properties, rather than the actual properties of the rhizosphere. In fact, these conductivities depend on the choice of the root length imposed in the model. We assumed that the two genotypes had the same root length (which was justified by the measurements; Supplementary Data Fig. S3b and c), but the genotype with higher mucilage production could have a larger fraction of the root surface active in water uptake, and this might well be the effect of mucilage keeping a larger fraction of the root system in hydraulic contact with the soil. The effect would still be an attenuation of the average flux at the root surface and of the gradients in water potential across the rhizosphere, but would not come exactly from the hydraulic conductivity curve shown in Fig. 5.

Mucilage has been shown to influence soil hydraulic properties and soil water dynamics on a macroscopic scale (Ahmed *et al.*, 2014; Carminati *et al.*, 2017*a*; Kroener *et al.*, 2018; Benard *et al.*, 2019). These impacts emerge from mucilage’s ability to alter the physical properties of the soil solution (Read and Gregory, 1997; Carminati *et al.*, 2017*a*; Naveed *et al.*, 2019; Benard *et al.*, 2021). Indeed, Benard *et al.* (2019) documented that a polymer network stretches during soil drying and forms an interconnected one- or two-dimensional matrix (Benard *et al.*, 2019). The formed structure increases water retention and liquid connectivity of the soil matrix (Benard *et al.*, 2019), which potentially bridges or even suppresses the formation of air gaps between the soil and root surface that may develop in dry soils (North and Nobel, 1997; Carminati *et al.*, 2009, 2013; Benard *et al.*, 2021). Note that in the study of Benard *et al.* (2019) no root system was involved, and the investigations focused on pore-scale mechanisms using artificial glass beads as an analogy of porous media to mimic how mucilage impacts rhizosphere hydraulics. We obtained our findings by observing juvenile plants under controlled conditions, which enabled a better understanding of fundamental mechanisms on a plant scale.

## CONCLUSION

We have demonstrated here that root mucilage significantly enhances root water uptake and maintains transpiration in cowpea plants. Notably, water potential decrease across the rhizosphere was attenuated by high mucilage production in moderately dry soils. These findings underscore the crucial role of mucilage in improving soil–root hydraulic conductance, thereby positively influencing plant water use regulation, and linearizing the relationship between transpiration rate and leaf water potential. Previous investigations into this phenomenon were limited to artificial set-ups and mathematical modelling due to technical constraints and the lack of appropriate plant material (Ahmed *et al.*, 2014; Kroener *et al.*, 2014; Carminati *et al.*, 2017*a*; Benard *et al.*, 2019; Hayat *et al.*, 2021; Rahim *et al.*, 2024). Unlike previous research, which did not involve plants, our work uniquely examines the role of mucilage in water uptake by measuring the dynamics of water potential and water fluxes across the soil–plant continuum during soil drying. In essence, mucilage markedly reduces the drop in matric potential at the root surface, facilitating a steady hydraulic supply from soil to root in transpiring plants.

These findings highlight how root mucilage production engineers rhizosphere hydraulic traits to sustain water fluxes from the soil and maintain transpiration during soil drying (Carminati *et al.*, 2010; Carminati and Vetterlein, 2013; Ahmed *et al.*, 2014; Benard *et al.*, 2019; Abdalla *et al.*, 2021). Based on our study, future research can now investigate the impacts of mucilage on water use regulation under natural field conditions and throughout the crop lifespan. We advocate for integrating below-ground and rhizosphere biophysical processes to illustrate how plants adapt to and mitigate the impacts of climate change.

## SUPPLEMENTARY DATA

Supplementary data are available at *Annals of Botany* online and consist of the following.

Figure S1: Soil hydraulic properties of loamy soil used in this study. Figure S2: Root pressure chamber system (RPCS) measures the relationship between transpiration (*E*) and leaf xylem water potential (*ψ*_leaf_). Figure S3: Representation of plant biomass of different genotypes. Figure S4: Segmented linear regression model results. Table S1: The parameters used in the soil–plant model. Notes S1: Soil–plant hydraulic model.

mcae193_suppl_Supplementary_Figure_S1

mcae193_suppl_Supplementary_Material

mcae193_suppl_Supplementary_Figure_S2

mcae193_suppl_Supplementary_Tables_S1

mcae193_suppl_Supplementary_Figure_S3

mcae193_suppl_Supplementary_Figure_S4

## Data Availability

The original contributions presented in the study are included in the article and the supporting information. For further inquiries please contact the corresponding author.
