# Supplementary material for: Mucilage facilitates root water uptake under edaphic stress: first evidence at the plant scale
Source: Ann Bot. 2024 Oct 30;136(5-6):987–96. doi: 10.1093/aob/mcae193 (PMC12682842; doi:10.1093/aob/mcae193)
Supplement: mcae193_suppl_Supplementary_Figure_S1 [file mcae193_suppl_supplementary_figure_s1.docx]

**
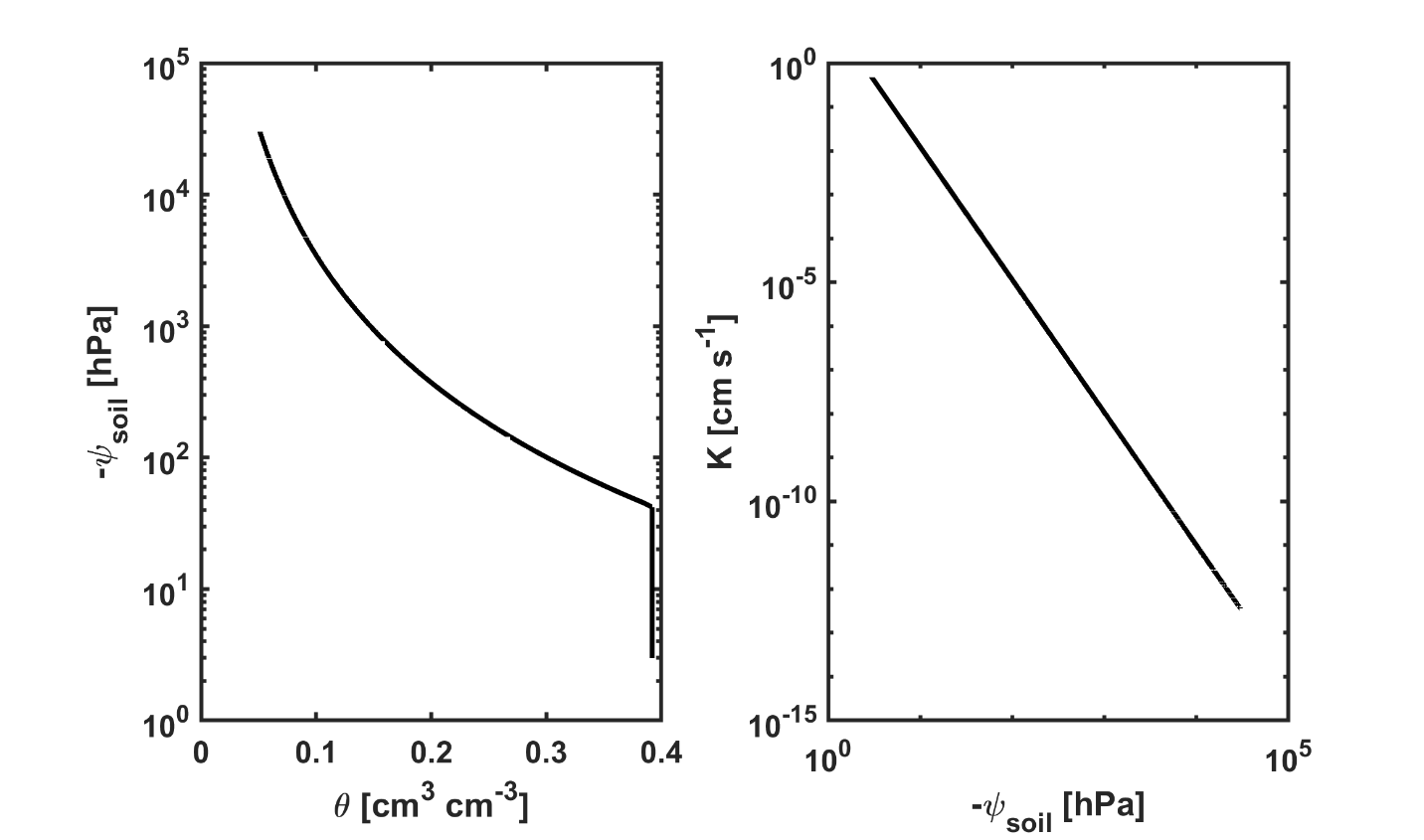
**

**Fig. S1.** Soil hydraulic properties of loamy soil used in this study. **a**) soil water retention curve (solid line) fitted by Brooks and Corey model that shows the relationship between soil water content (*θ*) and soil water potential (*ψ*_soil_). **b**) Soil hydraulic conductivity (*K*_s_) curve fitted by Brooks and Corey model for unplanted soil.
