# Supplementary material for: Mucilage facilitates root water uptake under edaphic stress: first evidence at the plant scale
Source: Ann Bot. 2024 Oct 30;136(5-6):987–96. doi: 10.1093/aob/mcae193 (PMC12682842; doi:10.1093/aob/mcae193)
Supplement: mcae193_suppl_Supplementary_Material [file mcae193_suppl_supplementary_material.docx]

**Supplementary Note S1: Soil–plant hydraulic model**

We used a soil–plant hydraulic model to simulate the water flow throughout the soil–plant system and to fit the relationship between transpiration rate (*E*) and leaf water potential (*ψ*_leaf_) during soil drying. Please note that the balancing pressure described in the Materials and Methods Section is numerically equal to *ψ_leaf_* (Abdalla *et al.*, 2021, 2022a). Water flow is modelled through a series of resistances across the soil, at the soil–root interface, across the root to the root xylem, and along the xylem. We briefly describe the model here.

The Buckingham–Darcy law was used to describe the radial water flow in soil toward the root surface:

$q=-K_{s}\left( \psi_{m} \right)\frac{\partial\psi_{m}}{\partial r}$ (Eqn S1)

where *q* is the water flux (cm s^−1^); *K_s_* is the soil hydraulic conductivity (cm^2^ s^−1^ hPa^−1^), which is a function of the soil matric potential $\psi_{m}$ (hPa); *r* is the radial distance (cm); and $\frac{\partial\psi_{m}}{\partial r}$ is the gradient in matric potential. Note that when the soil matric potential is expressed in unit heads (cm, 1 hPa ≈ 1 cm), *K*_s_ has units of (cm s^−1^). We used this unit throughout the text when describing soil water flow.

The boundary conditions were expressed as follows:

$q\left( r_{0} \right)=\frac{E}{2\pi r_{0}L}$ (Eqn S2)

$q\left( r_{b} \right)=0$ (Eqn S3)

where *r_0_* and *r_b_* are the root radius and the exterior radius of soil around the root (cm), *E* is the transpiration rate (cm^3^ s^−1^), and *L* is the root length active in water uptake (cm). *r_b_* is determined by *L* and the volume of the column V (cm^3^) according to:

$r_{b}= \sqrt{\frac{V}{\pi L}}$ (Eqn S4)

*K_s_* was parameterized according to the Brooks and Corey model:

$K_{s}\left( \psi_{m} \right)= K_{sat} {(\frac{\psi_{m}}{\psi_{0}})}^{\tau}$ (Eqn S5)

where *K_sat_* is the saturated hydraulic conductivity of the soil (cm s^−1^), $\psi_{0}$ is the soil air entry value (cm), and $\tau$ is a unit-less fitting parameter. Assuming a steady-rate behavior for the water flow in the soil, and according to de Jong van Lier *et al.* (2008), the radial geometry of water flow could be reformulated using the matric flux potential (*Ф*, cm^2^ s^−1^):

$\Phi\left( \psi\right)= \int_{-\infty}^{\psi_{m}} K\left( x \right)dx$ (Eqn S6)

The solution of Eqn 6 describes the matric flux potential at the outer boundary r = r_b_:

$\Phi_{soil}= \frac{k_{sat} . {\psi_{m}}^{1- \tau}. {\psi_{0}}^{-\tau}}{1- \tau}$ (Eqn S7)

$\Phi_{soil}$ is the matric flux potential in the bulk soil (cm^2^ s^−1^) corresponding to the measured bulk soil matric potential ($\psi_{m})$. Meanwhile, the radial flow could be described by combining Eqn1, 5, and 6 as:

$q=- \frac{\partial\emptyset(\psi_{m})}{\partial r}$ (Eqn S8)

We obtain the flux boundary condition at the soil–root interface, $\Phi_{root\_soil}$(cm^2^ s^-1^), by combining Eqn 5 and 6, and the radial Richards equation (Schröder *et al.*, 2009):

$\Phi_{root\_soil}= - \frac{E}{2\pi L}\left( \frac{1}{2}- r_{b}^{2} \frac{\ln\left( {r_{b}}/{r_{0}} \right)}{r_{b}^{2}- r_{0}^{2}} \right)+ \frac{k_{sat} . {\psi_{m}}^{1- \tau}. {\psi_{0}}^{-\tau}}{1- \tau}$ (Eqn S9)

The water flow in the root is given by

$E=-K_{root}\left( \psi_{root\_xylem}-\psi_{root\_soil} \right)$ (Eqn S10)

where *E* is the water flow in the root equal to the transpiration rate (cm^3^ s^−1^), $\psi_{root\_soil}$ is the water potential at the root–soil interface (here converted to MPa, where 1 MPa ≈ 10000 cm), $\psi_{root\_xylem}$ is the water potential at the xylem collar (MPa), and $K_{root}$ (cm^3^ s^−1^ MPa^−1^) is the root hydraulic conductance.

The xylem conductance, *K_x_* (cm^3^ s^−1^ MPa^−1^), which includes the effect of cavitation, is given by:

$K_{x}=K_{root}\left( \frac{\psi_{leaf}}{\psi_{0x}} \right)^{-\tau_{x}}$ (Eqn S11)

where *ψ_leaf_* is the leaf xylem pressure (MPa), ψ_0x_ is the xylem pressure (MPa) at which *K_x_* drops, and $\tau_{x}$ is a unit-less fitting parameter that determines the rate of this drop.

The plant conductance, *K_plant_* (cm^3^ s^−1^ MPa^−1^), is given by the harmonic mean of *K_root_* and *K_x_*:

$\frac{1}{K_{plant}}=\frac{1}{K_{root}}+\frac{1}{K_{x}}$ (Eqn S12)

The leaf matric flux potential is calculated as:

$\Phi_{leaf}= -E+ \Phi_{root\_xylem}$ (Eqn S13)

Leaf xylem pressure *ψ*_leaf_ is calculated by inserting *K_x_* into Eqn 6 and combining Eqn 11 and 13:

$\psi_{leaf} = (\psi_{root\_xylem}^{1 - \tau_{x}}- \frac{E (\tau_{x} - 1)}{\psi_{0x}^{\tau_{x}} K_{root}})^{\frac{1}{1 - \tau_{x}}}$ (Eqn S14)
