# Supplementary material for: Mucilage facilitates root water uptake under edaphic stress: first evidence at the plant scale
Source: Ann Bot. 2024 Oct 30;136(5-6):987–96. doi: 10.1093/aob/mcae193 (PMC12682842; doi:10.1093/aob/mcae193)
Supplement: mcae193_suppl_Supplementary_Figure_S2 [file mcae193_suppl_supplementary_figure_s2.docx]

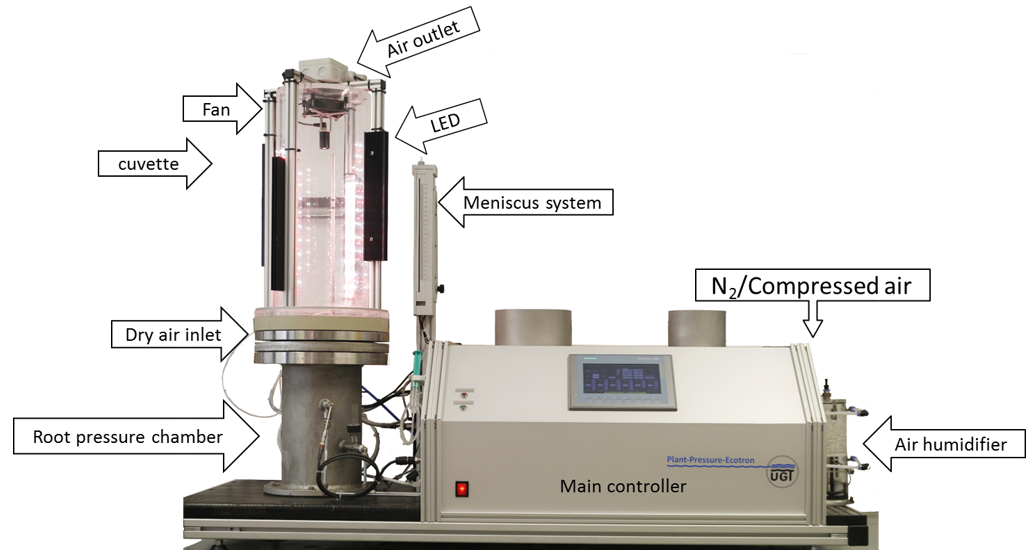


**Fig. S2.** Root pressure chamber system (RPCS) measures the relation between transpiration (*E*) and leaf xylem water potential (*ψ*_leaf_). Plants were placed inside the RPCS, with the column in the root pressure chamber and the shoot in the cuvette. *E* was altered by increasing the photosynthetic photon flux density (PPFD) stepwise. Pneumatic pressure was applied to balance the water pressure inside the leaf xylem at atmospheric pressure. The balancing pressure is numerically equal to the leaf xylem water potential (*ψ*_leaf_). *E* and *ψ*_leaf_ were simultaneously determined at each PPFD.
