# Supplementary material for: Mucilage facilitates root water uptake under edaphic stress: first evidence at the plant scale
Source: Ann Bot. 2024 Oct 30;136(5-6):987–96. doi: 10.1093/aob/mcae193 (PMC12682842; doi:10.1093/aob/mcae193)
Supplement: mcae193_suppl_Supplementary_Tables_S1 [file mcae193_suppl_supplementary_tables_s1.docx]

**Supplementary Table S1.** The parameters used in the soil-plant model.

| Parameter | Symbol | value | Unit |
| --- | --- | --- | --- |
| Soil saturated conductivity | *K_s_* | **CB** 5.58×10^-6^  **IT** 0.98×10^-6^ | cm s^-1^ |
| Fitting parameter for the unsaturated conductivity | $\tau$ | **CB** 1.96 | - |
|  |  | **IT** 1.46 | - |
| Root conductance | *K_root_* | **CB** 0.911×10^-6^  **IT** 1.87×10^-6^ | cm^3^ s^-1^ hPa^-1^ |
| Soil air entry value | $\psi_{0}$ | -8.33×10^-4^ | MPa |
| Xylem air entry value | $\psi_{0x}$ | -1.5 | MPa |
| Fitting parameter for xylem conductivity | $\tau$*_x_* | 5 | - |
| Active root length in water uptake | *L* | **CB** 51  **IT** 40 | cm |
| Root radius | r_0_ | 0.05 | cm |

***CB:** low root mucilage production; **IT:** high root mucilage production.
