# Supplementary material for: Mucilage facilitates root water uptake under edaphic stress: first evidence at the plant scale
Source: Ann Bot. 2024 Oct 30;136(5-6):987–96. doi: 10.1093/aob/mcae193 (PMC12682842; doi:10.1093/aob/mcae193)
Supplement: mcae193_suppl_Supplementary_Figure_S3 [file mcae193_suppl_supplementary_figure_s3.docx]

**
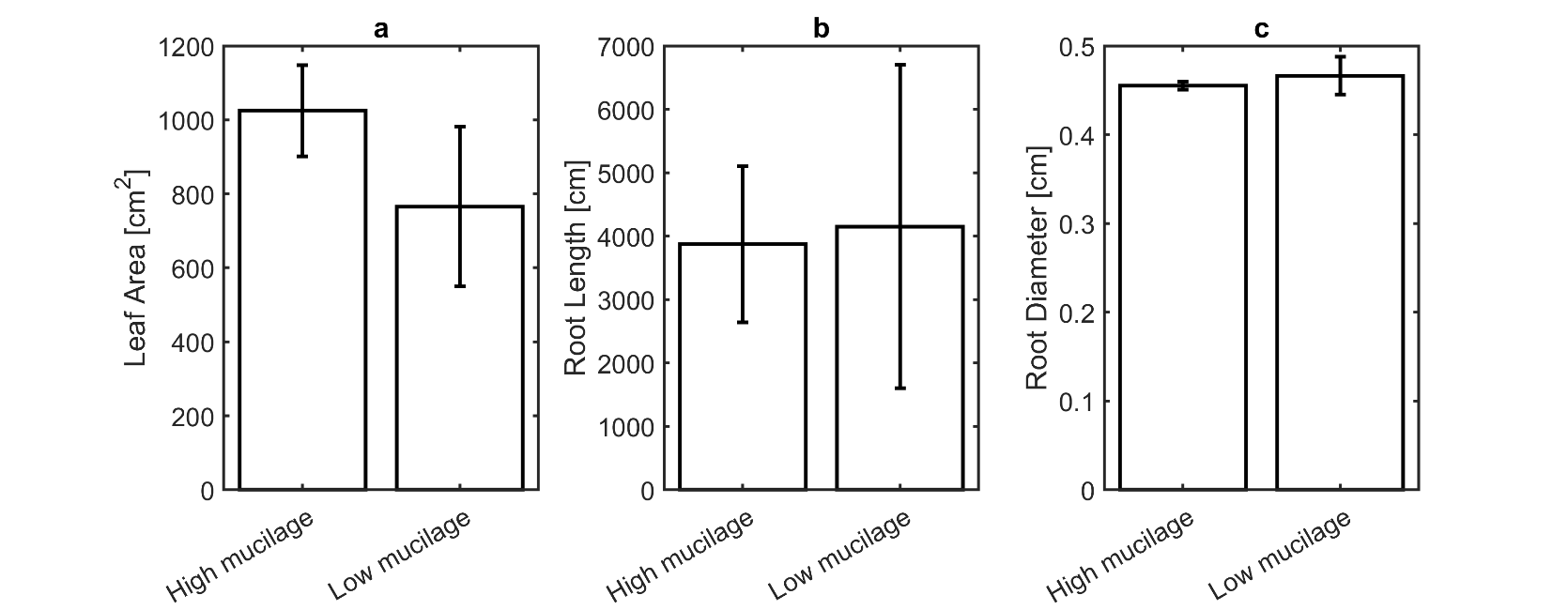
**

**Fig. S3.** Representation of plant biomass of different genotypes. **a**) Leaf area, **b**) root length and **c**) root diameter. Data represent the mean ± std. (N = 6). No significant differences were observed in leaf area (*p-value* = 0.0711), root length (*p-value* = 0.902), nor root diameter (*p-value* = 0.439).
