# Supplementary material for: Mucilage facilitates root water uptake under edaphic stress: first evidence at the plant scale
Source: Ann Bot. 2024 Oct 30;136(5-6):987–96. doi: 10.1093/aob/mcae193 (PMC12682842; doi:10.1093/aob/mcae193)
Supplement: mcae193_suppl_Supplementary_Figure_S4 [file mcae193_suppl_supplementary_figure_s4.docx]

**
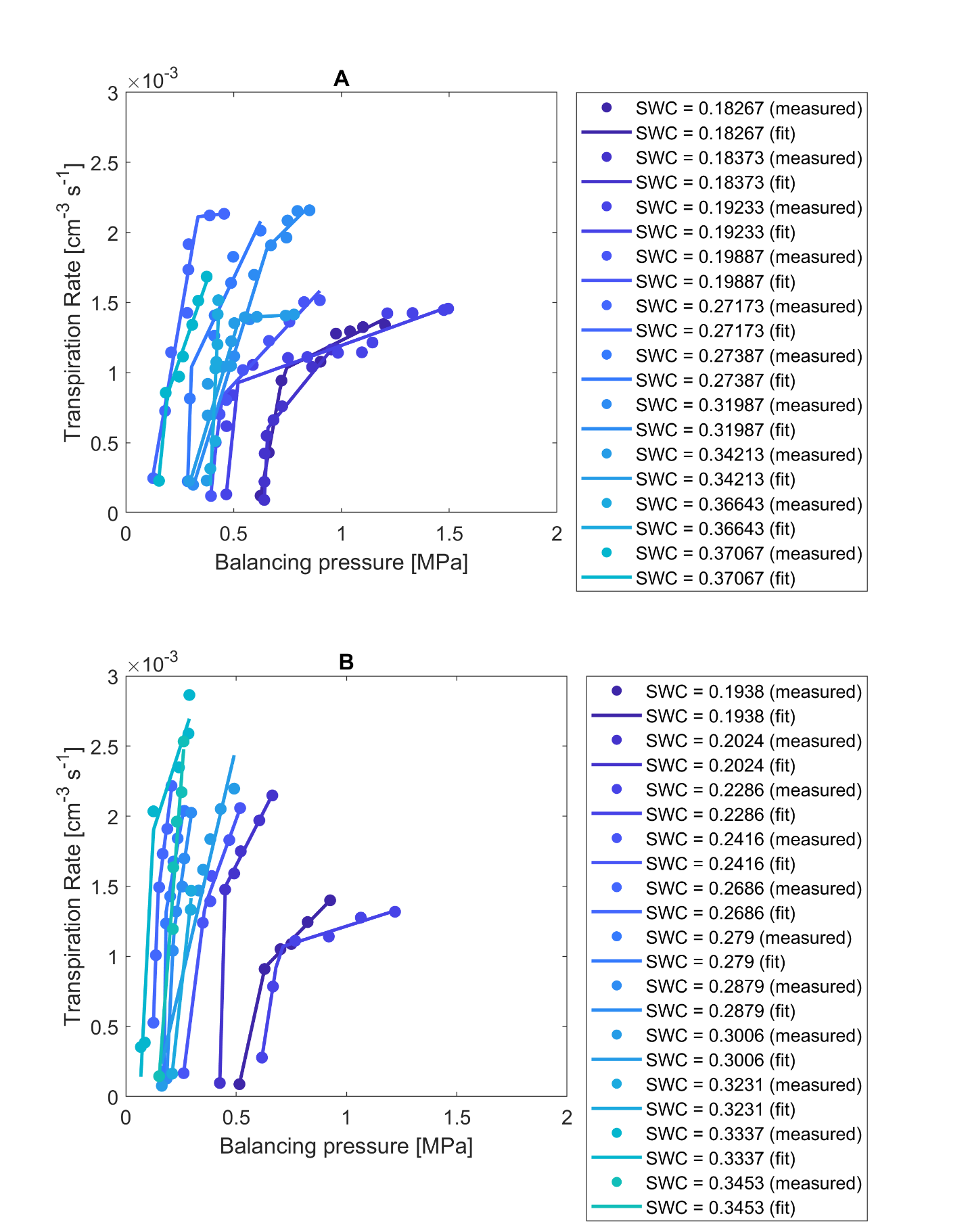
**

**Fig. S4.** Segmented linear regression model results. The model fitted the relationship between transpiration rate and balancing pressure at different soil water contents (SWC).
